# Stress-Induced Impairment of a Working Memory Task: Role of Spiking Rate and Spiking History Predicted Discharge

**DOI:** 10.1371/journal.pcbi.1002681

**Published:** 2012-09-13

**Authors:** David M. Devilbiss, Rick L. Jenison, Craig W. Berridge

**Affiliations:** University of Wisconsin-Madison, Madison, Wisconsin, United States of America; Indiana University, United States of America

## Abstract

Stress, pervasive in society, contributes to over half of all work place accidents a year and over time can contribute to a variety of psychiatric disorders including depression, schizophrenia, and post-traumatic stress disorder. Stress impairs higher cognitive processes, dependent on the prefrontal cortex (**PFC**) and that involve maintenance and integration of information over extended periods, including working memory and attention. Substantial evidence has demonstrated a relationship between patterns of PFC neuron spiking activity (action-potential discharge) and components of delayed-response tasks used to probe PFC-dependent cognitive function in rats and monkeys. During delay periods of these tasks, persistent spiking activity is posited to be essential for the maintenance of information for working memory and attention. However, the degree to which stress-induced impairment in PFC-dependent cognition involves changes in task-related spiking rates or the ability for PFC neurons to retain information over time remains unknown. In the current study, spiking activity was recorded from the medial PFC of rats performing a delayed-response task of working memory during acute noise stress (93 db). Spike history-predicted discharge (**SHPD**) for PFC neurons was quantified as a measure of the degree to which ongoing neuronal discharge can be predicted by past spiking activity and reflects the degree to which past information is retained by these neurons over time. We found that PFC neuron discharge is predicted by their past spiking patterns for nearly one second. Acute stress impaired SHPD, selectively during delay intervals of the task, and simultaneously impaired task performance. Despite the reduction in delay-related SHPD, stress increased delay-related spiking rates. These findings suggest that neural codes utilizing SHPD within PFC networks likely reflects an additional important neurophysiological mechanism for maintenance of past information over time. Stress-related impairment of this mechanism is posited to contribute to the cognition-impairing actions of stress.

## Author Summary

When faced with stressful situations, normal thought processes can be impaired including the ability to focus attention or make decisions requiring deep thought. These effects can result in accidents at the workplace and in combat, jeopardizing the lives of others. To date, the effect of stress on the way neurons communicate and represent cognitive functions is poorly understood. Differing theories have provided opposing predictions regarding the effects of stress-related chemical changes in the brain on neuronal activity of the prefrontal cortex (PFC). In this study, we show that stress increases the discharge rate of PFC neurons during planning and assessment phases of a task requiring the PFC. Additionally, using a point process model of neuronal activity we show that stress, nonetheless, impairs the ability of PFC neurons to retain representations of past events over time. Together these findings suggest that stress-related impairment of cognitive function may involve deficits in the ability of PFC neurons to retain information about past events beyond changes in neuronal firing rates. We believe that this advancement provides new insight into the neural codes of higher cognitive function that may lead to the development of novel treatments for stress-related diseases and conditions involving PFC-dependent cognitive impairment.

## Introduction

The prefrontal cortex (**PFC**) plays a central role in a diverse set of cognitive and behavioral processes, including sustained attention, working memory, and behavioral inhibition. In rat, the prelimbic region of the PFC (**_pl_PFC**) is a crucial subregion for these cognitive processes [1]–[4]. Delayed-response tasks of working memory have been extensively used to study the neurobiological basis of PFC-dependent function, in which information is retained during short delay intervals and used to guide subsequent behavior [5]–[11]. Seminal electrophysiological studies identified a subset of PFC neurons that display persistent spiking activity during delay periods of these tasks [7], [12]. Spiking rates during delay periods are correlated with both specific task-related cues and the number of cues required to be maintained during the delay period. Based on these observations, delay-related spiking activity is posited to reflect the maintenance of attentional processes, abstract rules, or past stimuli and events [13]–[15]. Additional evidence indicates that firing rates of PFC neurons during the response and reward phase of these tasks may reflect decision-related or reward/response outcome evaluation [12], [13], [16], [17].

Currently there are competing hypotheses in the literature regarding the potential effects of stress on PFC spiking activity. One view proposes that stress related increases in norepinephrine (**NE**) α_1_- and dopamine (**DA**) D_1_-receptor signaling within the PFC will act to inhibit persistent spiking rates during delay intervals of these tasks [18]. In contrast, it is also posited that stress-related increases in glucocorticoid-receptor signaling will enhance spiking rates by facilitating or increasing glutamatergic neurotransmission [19]–[22]. NE, DA, and glucocorticoids activate multiple receptor subtypes, each producing complex concentration- and receptor-dependent modulatory actions on spiking activity of target neurons [22]–[30]. Moreover, the combined actions of these neuromodulators on target neuron spiking rates during stress are difficult to predict. Indirect evidence also predicts that during stress, high levels of NE and DA may act to disconnect PFC neurons from excitatory recurrent feedback and suppress recursive, delay-related discharge of PFC neurons [18]. These actions are posited to involve the degradation of intrinsic neuronal mechanisms and excitatory recurrent neural connectivity that likely support the maintenance of information over time within PFC networks [2], [31]–[37].

Although there exists a large body of evidence demonstrating that stress impairs higher cognitive processes dependent on the PFC [38]–[40], surprisingly, to date the actions of stress on PFC neuronal discharge in animals engaged in tasks of working memory remain unknown. To address this gap in our understanding, we examined the relationship between acute noise stress-related impairment of performance during a PFC-dependent T-maze based delayed-response task of spatial working memory and stress-related changes in _pl_PFC neuronal spiking rates during the delay-period and other components of this task. Additionally, we directly determined the degree to which PFC neurons retain representations of past events over time by quantifying spike history-predicted discharge (**SHPD**) using a conditional intensity-generalized linear model statistical framework (**CI-GLM**) [41]–[45]. With the CI-GLM framework, we addressed several questions related to the actions of stress on PFC neuron function. First, to what degree does past spiking activity of PFC neurons predict or modulate ongoing activity of these neurons? Second, does stress have an overall impact on the predictability of PFC neuron discharge given a cell's intrinsic spiking history? Third, do specific task components (i.e. delay-period vs. behavioral response) interact with or modulate SHPD during baseline and acute stress? Combined, these studies represent a first characterization of the predictability of neural discharge from intrinsic spiking history within PFC networks of animals engaged in a cognitive task under normal and acute stress conditions.

## Results

### Characterization of Neural Recordings during a Working Memory Task

**Figure 1:**
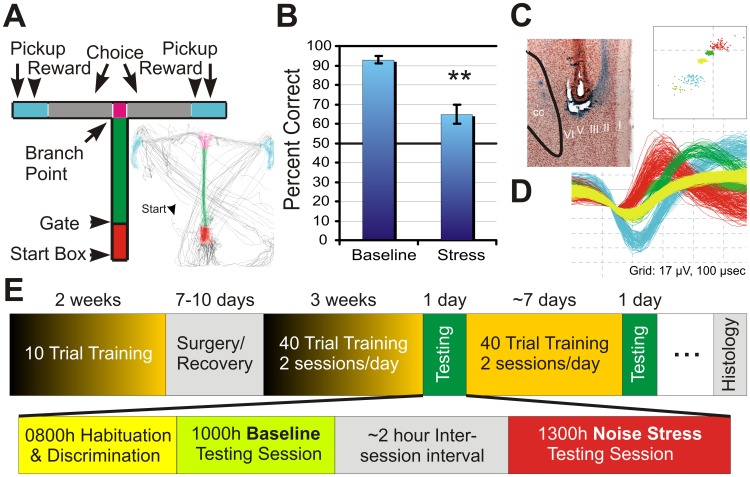
Electrophysiological recordings in rats performing the T-maze Task. **A**) Schematic of the T-Maze. On each trial, animals were placed in the start box (red) by the experimenter for a delay interval. The retaining gate was removed and the animal traveled to the branch point and revealed the choice to enter either arm (grey). Reward was then offered by the experimenter within the reward zone (cyan) if the arm opposite to the spatial location of last arm entered was chosen. Following reward or an incorrect choice, the animal was picked up by the experimenter and returned to the start box for a subsequent trial. **Inset** illustrates the video tracked path of a rat during one recording session. NOTE: paths crossing the T-maze from Pickup to Start Box reflect relocating the animal by the experimenter (right handed). **B**) Bar graph quantifying performance in the T-maze task during baseline and acute noise stress (93 db) conditions. n = 5; **p<0.01. **C**) 40× photomicrograph illustrating the final placement of one of eight microwires in layer V of the _pl_PFC (Arrow Tip; CC, corpus callosum; _pl_PFC, prelimbic PFC). **D**) Action potential waveforms of 5 discriminated and validated _pl_PFC neurons. Waveform width = 450 µs. Waveforms from these units exhibited separable clusters when plotted in principal component space (inset). Sorted spiking activity with unsorted activity is presented in Fig. S1. The cyan colored neuron represents the characteristic WS-type neuron. **E**) Timeline of behavioral training and testing. Details of a single testing day are shown beginning at 8:00 AM.

Five animals were tested in a T-maze based delayed-response task of spatial working memory (**Fig. 1** **and Fig. S2**). This task has been previously shown to be dependent on a functionally intact PFC and require PFC-dependent cognitive processes [4], [24], [30], [38]. In this task, animals were required to enter the T-maze arm opposite from the one last visited, following a delay period to obtain food rewards (chocolate chips 1.6 gm) delivered by the experimenter's hand. Delay length was specific to each animal (range 10–40 sec) and chosen to maintain baseline performance near 90% correct. Animals were tested for two sessions a day separated by two hours during which the animal remained tethered to the recording equipment. Each session consisted of 41 trials; the 1^st^ trial of each session was always rewarded and not analyzed. During the first session (baseline), low-level masking white noise (60 db) was presented continuously and animals performed well (average 93% accuracy) with 3–4 errors occurring sporadically throughout the session (**Fig. 1B**). Intense white noise (93 db), presented throughout the second testing session, significantly impaired performance in this task, with animals performing at an average of 64.8% (−28%; p<0.0005 pairwise t-test). Acute white noise is a well-characterized stressor that elicits the physiological responses of stress and impairs PFC-dependent cognitive function in both humans and animals [18], [40], [46]–[51].

In these same animals, bilateral implants of electrode arrays permitted simultaneous recordings of extracellular discharge activity from layer V of the _pl_PFC yielding 491 spike trains from single neurons (**Fig. 1C–D**) [46], [52]. A subset of 339 of these neurons were classified as “wide spike” (**WS–type**) based upon action potential features, thus putative glutamatergic pyramidal neurons [53], and exclusively used for these analyses. Analyses of spiking activity were limited to trials containing correct responses, given so few error trials occurred during baseline testing sessions and reliable estimates of neuronal spiking activity were difficult to obtain.

### Effects of Stress on Delay- and Response-Related Discharge Rates in the plPFC

**Figure 2:**
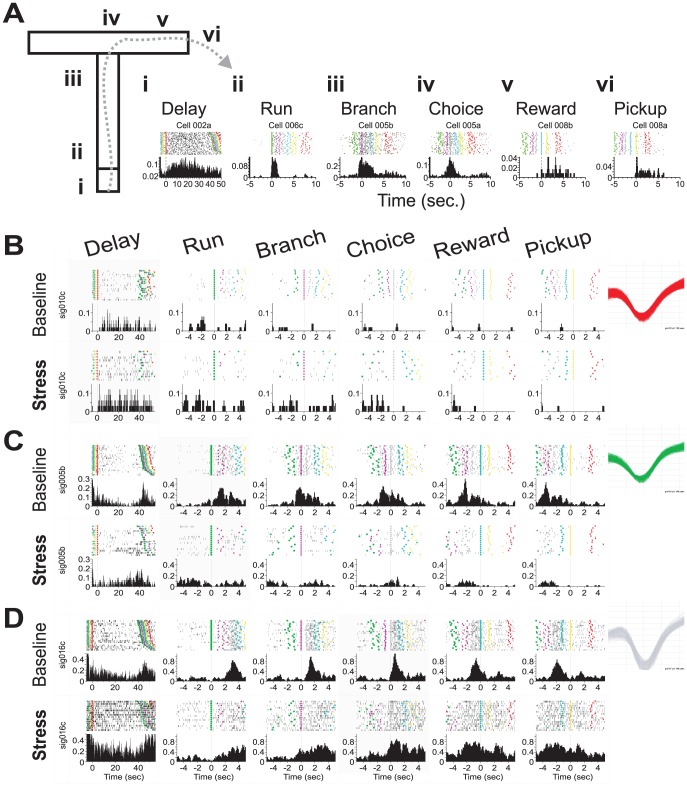
Peri-stimulus time histograms (PSTHs) illustrate the effects of stress on _pl_PFC neuron task-related spiking activity. **A**) T-maze schematic and associated peri-event raster and histogram analysis illustrates prototypic (i) delay- (ii) run- (iii) branch- (iv) choice- (v) reward- and (vi) pickup-related activity observed from _pl_PFC neurons (0 sec. = start of respective behavioral interval; n = 40 correct trials of a baseline recording session; Delay length = 20 sec.; 5 msec. bins). Colored fiduciaries indicate beginning of each major event of the T-maze task (Red, Start Box; Green, Gate; Magenta, Branch; Grey, Choice; Cyan, Reward; Yellow, Pickup). **B**) Task-related discharge of a single WS-type _pl_PFC neuron during correctly executed trials with a left arm entry during the baseline recording session (17 trials; top) and subsequent stress session (11 trials; bottom). Delay-related spiking of this delay neuron was enhanced during stress. Inset illustrates recorded spike waveforms. PSTH y-axis represents spiking probability/bin normalizing for different numbers of trials (5 msec. bins). **C**) Run-related activity suppressed during stress conditions. **D**) Suppression of choice-related activity during stress. Labeling conventions of C–D are identical to B.

Discharge rates of WS-type _pl_PFC neurons were characterized for intervals of the T-maze task (e.g. delay, run, and reward) using a peri-event time histogram (**PETH**) approach. Under baseline conditions, the discharge rate of _pl_PFC neurons fluctuated throughout the time-course of each trial. The pattern of spiking activity was neuron-specific, with neurons exhibiting selective increases in discharge rate during single or adjacent behavioral intervals. A significantly large number of _pl_PFC neurons exhibited delay-related spiking activity during baseline conditions (48.7%; χ^2^ = 21.4, p<0.001). During delay periods, the discharge rates of these neurons averaged 0.55 Hz+/−0.047 SEM. The illustrative cases shown in **Fig. 2A** demonstrate task-related fluctuations in discharge rate corresponding to delay periods, components of the behavioral response, and reward intervals across the recorded population of _pl_PFC neurons during baseline sessions.

**Figure 3:**
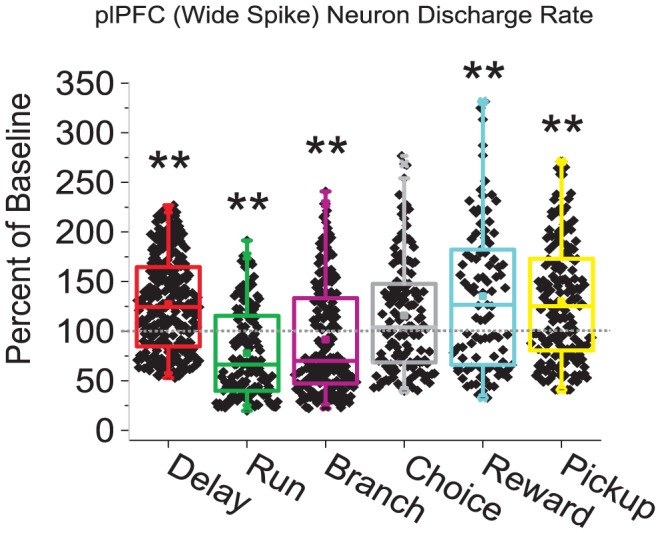
Effects of stress on task-related discharge rates of _pl_PFC neurons. Average discharge rates of WS-type _pl_PFC neurons during stress conditions were quantified for each behavioral interval of correct trials and plotted as a percent change from baseline conditions (1^st^ recording session) for matched behavioral intervals trials with identical T-maze arm choices. Box and whisker plots illustrate that during stress, discharge rates within the Delay, Reward, and Pickup behavioral intervals are increased. During the Run and Branch behavioral intervals, discharge rates are suppressed under stress conditions. Colored box and whiskers designate the first and fourth quartiles and median line (box), distribution mean (dot), and 5–95% range of the data (whiskers). (**p<0.01 FDR corrected T-Test compared to baseline).

As illustrated by the examples shown in **Fig. 2B–D**, the effects of stress on _pl_PFC neuron spiking rates were dependent on the behavioral intervals of the T-maze task. For these cases, stress increased delay-related activity 180% of baseline (0.018 to 0.033 Hz; **Fig. 2B**), whereas response related activity was suppressed during the run phase (42% of baseline, 0.092 to 0.039 Hz; **Fig. 2C**) with little effect during the choice phase (108% of baseline, 0.748 to 0.811 Hz; **Fig. 2D**). These opposing actions of stress on delay-related activity versus response-related activity were frequently observed across simultaneously recorded neurons within an animal. Within each component of the task, stress produced cell-specific modulatory actions on spiking rate of _pl_PFC neurons, similar to prior reports on catecholamine neuromodulatory effects [25], [29], [52]. Nonetheless, across all recorded WS-type _pl_PFC neurons, stress significantly affected task-related spiking rates of correct trials (rmANOVA(TaskComponent) F_(5,3810)_ = 579.05, p<0.0001). During delay-periods, stress significantly *increased* the average discharge rate of these neurons (127% of baseline, **Fig. 3**). In contrast, stress *suppressed* the average spiking activity during the run and branch components of the behavioral response (78% and 92% of baseline respectively). During choice intervals, stress produced a modest increase in the average discharge rate (115% of baseline) but this effect was not statistically significant. Lastly, similar to that seen during the delay period, PFC neuron discharge rates during the reward and pickup components of the task were also facilitated by stress (135% and 130% of baseline). No differences were observed between the effects of stress on right vs. left trials. As such, these data suggest that intense acute stress generally enhances delay-related activity across PFC neurons. Moreover, the combined actions of acute stress across task intervals support the hypothesis that stress could differentially affect PFC-dependent processes that occur during the delay versus response period of these tasks.

### Effects of Stress on Spike History-Predicted Discharge of _pl_PFC Neurons

PFC neuron spiking activity during the T-maze task was further studied using CI-GLM's to assess whether SHPD significantly contributed to the ongoing activity of PFC neurons and the degree to which acute noise stress altered the predictability of PFC neuron discharge given a cell's intrinsic spiking history. The CI-GLM model **(1a)**,
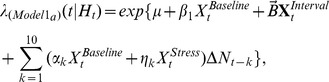
included covariates representing the background level of spiking activity (intercept; *μ*), an index of baseline and auditory stress conditions within a testing day 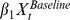, the T-maze behavioral intervals (delay, run, branch, choice, reward, and pickup) 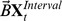, and a tenth order autoregressive process during baseline as well as during noise stress conditions 
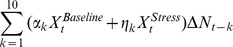
 with discretized time for increasing spike history durations represented as 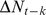. Detailed descriptions of this and subsequent equations are presented in the Materials and Methods. From this model, the “spiking gain” of predicted discharge activity during baseline (*α*) and stress (*η*) conditions was calculated for each 250 ms bin back in time. Explicitly, the spiking gain is equivalent to the rate-ratio (exponentiated covariate weights i.e. *α, η*) [54] and represents the fold-change in predicted discharge activity given all other spiking activity occurring during the T-maze task.

**Figure 4:**
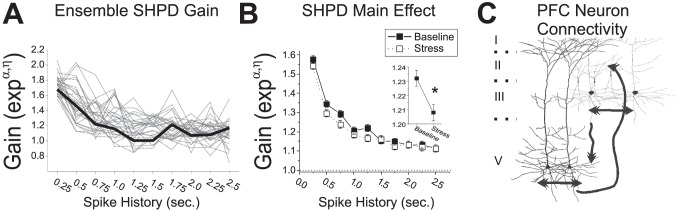
Stress-related changes in _pl_PFC neuron spike-history predicted discharge (SHPD) throughout baseline or acute stress conditions. **A**) Spiking gain (rate-ratio exp*^α,η^*) measures of the contribution of spike history at different points back in time for a small (n = 50) ensemble of neurons. The SHPD gain of one exemplar neuron is highlighted. **B**) SHPD gain at different points back in time decay exponentially under baseline and stress conditions (Model 1a). Described in the main text, stress produced an overall reduction in SHPD gains (inset; *p<0.005), but did not significantly alter the decay of gains at any spike history time bin. **C**) Schematic of recurrent pathways within the PFC of connectivity within layers II/III or V as well as connectivity between II/III and V represents one putative mechanism supporting SHPD. Adopted from: [72]–[74].

Individual _pl_PFC neurons exhibited unique patterns of SHPD for each point back in time. For the illustrative cases shown in **Fig. 4A**, SHPD gains generally decayed with increasing time points in the past. Nonetheless, _pl_PFC neurons frequently demonstrated non-monotonic changes in SHPD gains at specific time points. For example, the highlighted pattern of SHPD gains of a single _pl_PFC neuron illustrates both a decay in SHPD gains over time and a selective increase in SHPD gain at 1.75 seconds. Similar to these individual cases the pattern of SHPD gains, averaged across the recorded population of WS-type neurons, decayed exponentially with increasing points further back in time during baseline conditions (f_(x)_ = 1.16+0.544^(−x/0.25)+0.51^; **Fig. 4B**). Furthermore, although SHPD gains decayed over time, gains remained significantly greater than 1.0, indicating that spike history positively predicted future discharge for at least 2.5 seconds. Interestingly, the overall pattern of SHPD gains observed from _pl_PFC neurons differed from other areas of the cortex where SHPD does not significantly modulate ongoing spiking activity after 100 ms [55]. This difference may reflect unique intrinsic neuronal or circuit properties of the PFC. Although acute noise stress did not alter the pattern of SHPD gains over time (ANOVA(Stress*Time) F_(9,3523)_ = 0.86, p = 0.560), stress did significantly reduce the magnitude of SHPD gains across all time intervals tested (ANOVA(Stress) F_(1,3523)_ = 8.78, p = 0.0031; **Fig. 4B** **inset**).

To confirm that task-related fluctuations in spiking rates were important to include in these models, Model 1a can be compared to the reduced model lacking T-maze behavior intervals covariates (i.e. 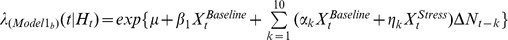). A comparison of these two models for each _pl_PFC neuron, demonstrated that the measure of deviance was significantly reduced for the majority of neurons (85.7%, 187 of 218 neurons; χ^2^ test, FDR corrected p value <0.0113) when CI-GLM's included the T-maze behavior intervals (Model 1_a_). When analyses were replicated with the reduced model, a similar stressor-induced significant suppression of SHPD was found across individual _pl_PFC neurons (ANOVA(Stress) F_(1,4448)_ = 19.71, p<0.0001; **Fig. S1**). Together these results support the general hypothesis that, in addition to task-related fluctuations in spiking rates, spike history plays an important role in shaping _pl_PFC neural discharge. Furthermore, the fact that stress suppressed SHPD in animals performing the PFC-dependent T-maze task likely suggests that SHPD may be an important mechanism supporting PFC-dependent cognitive functions. However, the degree to which these effects are specific to the T-maze task remains to be determined. Reverberations in recurrent cortical circuitry (**Fig. 4C**) or intrinsic neuronal mechanisms likely contribute to SHPD [31], [35], [39], [56] and permit PFC neurons to maintain and preserve information within spiking patterns across large time intervals. However, the fact that stress did not alter the pattern of decay for SHPD gains throughout each trial suggests that stress may not change the underlying mechanism(s) generating SHPD.

### Spike History-Predicted Discharge Is Selectively Impaired during Delay Intervals

**Figure 5:**
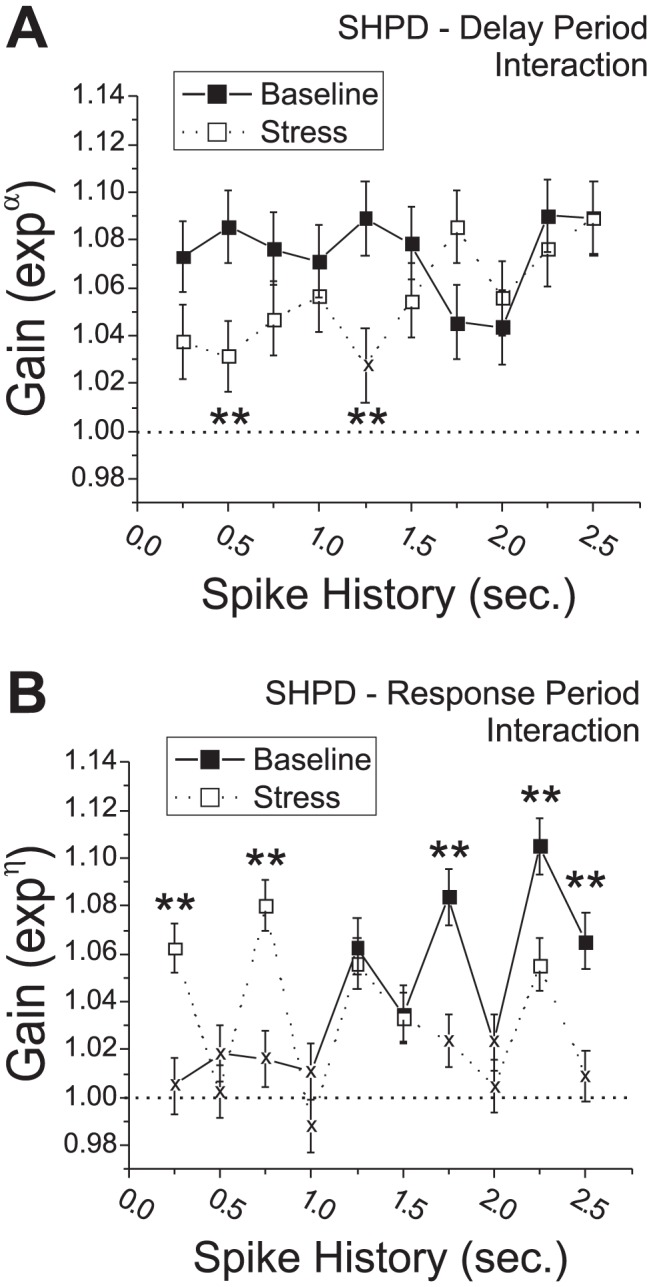
Stress-related changes in delay- and response-related _pl_PFC neuron spike-history predicted discharge (SHPD). **A**) Delay-specific gains of SHPD interaction terms from the CI-GLM (Model 2; *α, η*) were averaged across _pl_PFC neurons and plotted. **B**) Response interval-specific gains of SHPD interaction terms (Model 3). Stress suppressed delay-specific SHPD gains and increased the impact of the most recent spiking history during the response period. (*p<0.05 FDR corrected compared to baseline; x<0.05 FDR corrected compared to 1.0).

Delay-related spiking activity is posited to serve a pivotal role in the accurate performance of delayed response tasks of working memory [2], [3], [18], [30], [57]. To determine if stress preferentially affects SHPD during delay intervals, a second CI-CLM was formulated to examine the interaction between spike history and the extended delay interval (Pickup-Delay; **Model 2**). 
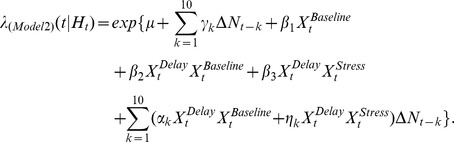
This model included the intercept (*μ*), the main effects of SHPD 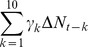, baseline and stress conditions within a testing day 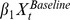, and the first-order extended delay interval interaction 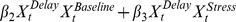. The second-order interaction terms of this model, 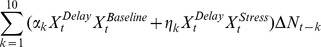, correspond to delay-specific SHPD during baseline (α) or stress (*η*) conditions beyond the main effects of stress accounted for in Models 1a,b. During baseline conditions, the gains of delay-specific, SHPD (*α*) averaged across all WS-type _pl_PFC neurons were all positive and significantly greater than one (**Fig. 5A** **)**. For the first 1.5 seconds of spiking history, gains were essentially flat and averaged 1.08+/−0.0045 (SEM). During acute noise stress, gains for the most recent spike history intervals (*η*) were suppressed from baseline levels (ANOVA(Stress*Time) F_(9,4671)_ = 2.00, p = 0.035). Although stress-related suppression of delay-specific, SHPD gains was observed for intervals up to 1.5 seconds, a statistically significant difference from baseline levels was only observed at 0.5 and 1.25 seconds (FDR corrected). Moreover, delay-specific SHPD gains at the 1.25 second time point were not statistically different than 1.0 during conditions of noise stress. Given that the comparison between baseline and stress conditions was determined in the same CI-GLM, no deviance tests were performed. We posit that suppression of delay-specific SHPD of PFC neurons during conditions of stress contributes to stress-dependent impairment in delay-related PFC-dependent functions.

We next examined the interaction between spike history and the response interval (Run-Branch-Choice; **Model 3**):
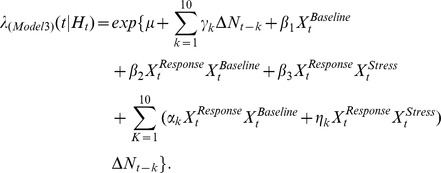
Similar to Model 2, gains associated with these model interaction terms represent response-specific gain of SHPD beyond the main effects of stress accounted for in Models 1a,b. Response-specific interaction term gains exhibited several important stress-related effects, even though these effects were more complex than the effects of stress on delay-specific interaction gains. First, during baseline recordings, response-related gains for intervals up to 1.0 second were not significantly different from one, making no contribution to the prediction of discharge activity (**Fig. 5B**). Stress significantly *increased* these response-specific SHPD gains at 0.25 and 0.75 second intervals (ANOVA(Stress*Time) F_(9,6894)_ = 7.3, p<0.0001). Second, under baseline conditions spike history response-related gains at longer intervals increased gradually to become significantly different than one. Stress significantly reduced long spike history interval response-specific gains to values that were equivalent to one (1.75, 2.0, and 2.5 seconds). We posit that the different effects of stress, on delay-specific versus response-specific gain of SHPD, likely reflect differing roles for SHPD in the cognitive or behavioral processes that occur during these behavioral intervals.

## Discussion

The present study characterized the actions of acute noise stress on discharge rate and SHPD of medial PFC neurons in rats engaged in a T-maze delayed-response task. This stressor impaired performance in this task and generally suppressed the ability for past spiking activity of PFC neurons to predict or modulate these neurons ongoing activity. We further demonstrate that the effects of stress on SHPD as well as discharge rate are dependent on specific phases of the task including the delay and response periods. During delay periods, stress suppressed SHPD of _pl_PFC neurons and enhanced delay-related firing rates. Outside of the delay period, SHPD was increased during the response period associated with a suppression of spiking activity during these same conditions of stress. These observations begin to provide an important link between the well-documented effects of stress on PFC-dependent cognitive functions and the impact of stress on PFC neural codes during a delayed-response task used to probe PFC-dependent function. Combined, these studies identify broad effects of stress on PFC neuronal activity that likely represent key aspects of the neurophysiological bases of stress-related cognitive impairment.

### Technical Considerations

The T-maze delayed alternation task embodies a number of important cognitive/behavioral processes and neurophysiological features associated with PFC function of human and non-human primates. This task is highly dependent on the PFC and sensitive to the effects of stress [30], similar to tasks used in humans to probe PFC function. Additionally, it is posited that the T-maze task requires PFC-dependent processes including working memory, attention, inhibition of proactive interference, and inhibition of distracter interference (generated by handling the animal between each trial), similar to tasks used to probe PFC-dependent function in primates and humans [1], [33], [58], [59]. In animals performing the T-maze task, we observed a significant number of PFC neurons exhibiting delay-related spiking activity, similar to that observed in primates performing delayed-response tasks [33], [60], [61]. More automated versions of these tasks, including the Figure-8 maze task, lack distracter interference and possibly other processes requiring significant engagement of the PFC. Such differences between the T-maze task and Figure-8 tasks may explain why automated delayed-response tasks have shown only few delay-related cells in rodent PFC [62].

Intense white noise is a well-characterized audiogenic stressor that impairs working memory, attention, and other PFC-dependent functions in rats, monkeys, and humans [38], [48], [51], [57], [63]. In the current study, continuous presentation of intense white noise (93 db) impaired performance of the T-maze based delayed-response task of spatial working memory and increased PFC neuron firing rates during the delay period. Similar impairment in T-maze performance is seen when animals are exposed to restraint stress *immediately prior* to testing [64], [65], but not after 4 hours of recovery from restraint stress [22]. Although previous studies have demonstrated that non-stressful white noise can activate PFC neurons [66], these cells are few in number (approximately 2% of PFC neurons). For these PFC neurons, responses to white noise are phasic, quickly adapting, and are linked to the onset of the stimuli. During presentation of noise stress in the current study, PFC neuron firing rates were increased during the delay period but, importantly, were suppressed during the behavioral response. Together, the above observations provide strong evidence that the effects of noise stress on PFC neuron spiking activity were not induced by a continued sensory response to intense white noise.

In the present study, a CI-GLM framework was used to examine the degree to which past spiking activity of _pl_PFC neurons contributes to ongoing neural discharge patterns. Although the CI-GLM approach has been used successfully to distinguish between intrinsic spiking-history related discharge and extrinsic activity in motor cortex [67], [68], here we extend its use to examine SHPD in PFC networks. An advantage of this approach over peri-event time histogram analysis or other univariate analyses, including autocorrelegram analysis, is that the CI-GLM approach can disambiguate the relative contributions of spiking history from that of experimental and task-related variables to spiking activity. In the current study, task- and stress-related changes in overall firing rates were captured in the *β/B* terms of the model separately from the effects of the interaction terms (*α* or *η*). Thus, SHPD during baseline could be directly compared to SHPD during stress conditions in a manner that accounted for the effects of stress on the overall discharge rate and task-related fluctuations in spiking activity within each trial. A second consideration for the CI-GLM approach is the use of a Poisson distribution to fit the CI-GLM to neural data over other distributions, including Gaussian or Bernoulli. By doing so, this does not imply that a Poisson process generates _pl_PFC neuronal spiking activity. Instead, the Poisson distribution is the appropriate distribution for what is, in simplified terms, a spiking count-based multivariate regression and provides a computationally tractable solution to fit _pl_PFC neuronal spiking activity. A number of excellent reviews described these statistical modeling methods and the appropriate use of these models to characterize spike trains (e.g. [45]).

### The Role of Spike History in Cognitive Function

The present findings demonstrate that under baseline conditions, past spiking activity of a _pl_PFC neuron positively predicts future neuronal discharge. The contribution of past spiking activity to ongoing discharge of _pl_PFC neurons decayed exponentially with time during baseline and stress conditions; spiking-history at time points up to 1–1.5 seconds comprised most of the predictive power. With delay periods ranging from of 10–40 seconds and behavioral responses lasting several seconds, we posit that the time scale of SHPD is likely an important PFC neural process for the stable maintenance of information for short intervals during delay periods of working memory tasks while providing a mechanism that allows integration of new spiking activity patterns within PFC networks that permits flexible goal-directed behaviors. This time scale and pattern of decay is an order of magnitude longer to what has been observed in primary motor cortex [67], [68]. In the motor cortex, SHPD does not contribute to the ongoing activity of those neurons after 100 ms and reflects the refractory and recovery periods of those motor neurons. Thus, these differences likely reflect the excitatory recurrent network connectivity of PFC networks and the time scale on which these cortical networks must maintain information to perform their requisite neurocomputations. Furthermore, the current results suggest that neural codes involving SHPD are likely a complement to firing rate-based codes within the PFC. We found that CI-GLMs which account for modulation in firing rates across different task intervals were significantly better at modeling the spiking activity of the neurons. Moreover, SHPD gains were reduced by approximately 10% when the behavioral intervals are included as CI-GLM covariates, suggesting that both SHPD and components of the task account for the variance in spiking activity.

The current study also extends the idea that excitatory recursive activity within the PFC may be critical to sustain spiking activity and may act as the neurobiological basis of working memory and/or other cognitive processes occurring during these tasks [15], [69]–[71]. Early anatomical and recent computer models have suggested that recurrent neural connections within layers II/III and V of the PFC may support recursive, sustained activity during delay periods of working memory tasks [31]–[35], [72]–[74]. Together, those studies concluded that sustained discharge generated by reverberating excitatory feedback among anatomically connected networks of neurons during delay periods is the realization of maintaining past stimuli and events, attentional processes, and abstract task rules [13]–[15], [18], [75]. However, a large body of research further implicates AMPA and NMDA receptors and intrinsic calcium-dependent mechanisms in the generation and preservation of delay-related sustained discharge [56]. Specifically, the decay of NMDA currents are generally in the range of >80 ms, but some components require seconds to decay [76], suggesting that NMDA channels and associated maintenance of excitatory currents could explain the maintenance of delay-related sustained discharge. Thus, although the current study confirms that spiking history contributes to PFC neuronal discharge activity, the precise network or cellular mechanisms underlying SHPD remains to be identified. Moreover, the timescale of SHPD for _pl_PFC neurons could result from a combination of intracellular intrinsic calcium-dependent mechanisms [56], [69], [76]–[78], recurrent excitatory connections between neighboring neurons [13], [32], [34], as well as long recurrent paths between other cortical and subcortical brain regions, which remains to be tested.

### The Effects of Stress on PFC Neural Activity

The current study found that acute noise stress increased medial PFC neuron discharge rates during delay periods of rats performing a T-maze task of spatial working memory. In contrast, a number of prior observations predicted that during stress, high levels of extracellular catecholamines were likely to result in a suppression delay related firing rates. During acute stress, NE and DA neurotransmission in the PFC is elevated, activating low-affinity noradrenergic α_1_- and β-receptors as well as the dopamine D_1_-receptor [23], [26], [27], [30]. Activation of α_1_ receptors in the PFC has been shown to suppress delay-related sustained discharge to specific cued target directions during working memory tasks [24]. Similarly, high levels of D_1_ activation in the PFC suppresses delay-related activity to cued and non-cued target directions [29]. Extensive evidence suggests that the discrepancy between this prior prediction and the current observations is most likely due to stress-related increases in glutamate/glucocorticoid signaling within the PFC during acute stress [19]–[22]. Nonetheless, stress increases signaling within the PFC of a number of neuromodulators that may also contribute to changes in PFC neural activity during stress [79]. Increased delay-related discharge rates could reflect that, during stress, competing patterns of activation are instantiated across PFC neural populations during delay-periods that contribute to a disruption of working memory and sustained attention. Such interpretation of these data is supported by recent findings that a stress-related peptide, corticotrophin releasing factor, and activation of α_1_- receptors likely facilitates behavioral flexibility and processes involving attentional shifting [79], [80].

Although delay-related neuronal discharge rates are an important measure of PFC function, neural codes involving SHPD are also likely important for computations driving cognitive/behavioral processes in a number of cortical regions, including the PFC. In the current study, we demonstrate that stress impairs SHPD, principally during delay periods. These observations are consistent with theories that stress likely impairs the ability of PFC neurons/networks to continually update and maintain information necessary for appropriate behavioral responses through the delay period [7], [13], [32], [34], [36], [56], [81]. For example, it has been hypothesized that stress-related increases in NE and DA neurotransmission within the PFC act to modulate intracellular cyclic adenosine monophosphate signaling and hyperpolarization-activated cyclic nucleotide-gated channel function thereby disconnecting PFC neurons from excitatory recurrent feedback [18]. Thus, regulation of recurrent excitatory activation within PFC recurrent circuitry [31], [39] or regulation of NMDA related intracellular mechanisms [21], [35] may underlie the current findings that stress impairs SHPD and the ability of _pl_PFC neurons to retain representations of past events over time.

Lastly, we found that during the response phase of the task, firing rates of PFC neurons were suppressed during noise stress. Such actions are likely important for the complex repertoire of effects of acute stress across a range of cognitive functions. Suppression of PFC activity during the behavioral response could suggest that during stress, the PFC fails to appropriately inhibit behaviors mediated by other brain regions such as the dorsomedial striatum [82]. Such a loss of behavioral inhibition is supported by observations that habit-based actions, requiring little working memory, are favored under conditions of acute stress [40]. Furthermore, these data also support recent findings that rats prefer choices in decision-making tasks that require the least amount of work for reward following acute stress [83]. During the response phase of the task, SHPD interaction gains were enhanced at short intervals and suppressed at long intervals during acute stress. Such enhancement of short interval gains may reflect PFC activity patterns generated in the absence of ongoing inputs that could result in uncertainty/ambiguity of goal selection [84]. Additionally, it is possible that suppression of SHPD generated from spiking events in the distant past (i.e. >1.5 seconds) by stress could reflect impairment of the maintenance of information from the delay period into the response interval. Brain regions outside the PFC, including the dorsomedial striatum, could use PFC neural codes involving SHPD to guide selection of appropriate behavioral responses towards a rewarded goal. Nonetheless, the current findings are highly consistent with studies demonstrating that acute stress impairs cognitive functions requiring the PFC, whereas functions not dependent on the PFC such as hippocampal- and amygdala-related processes may be facilitated with stress [18].

### Summary

In summary, these results provide the first evidence that stress impairs the ability of past spiking activity to predict or modulate ongoing activity within PFC neuronal networks during delay periods of working memory tasks. Regardless of whether spike history-predicted discharge reflects an intracellular [18], [35] or neural circuit mechanism [31], [39], the current observations suggest that stress impairs the ability of PFC neurons/networks to continually update and maintain information through the delay period likely necessary for appropriate behavioral responses. Outside of the delay period, we conjecture that stress-related changes in spike history gain during the response period could represent inappropriate network reactivation related to an inappropriate goal selection [84], [85] or uncertainty/ambiguity of goal selection. Combined, these studies identify broad effects of stress on PFC neuronal activity that likely represent a key aspect of the neurophysiological bases of stress-related cognitive impairment.

## Materials and Methods

### Animals

Five male Sprague-Dawley rats (300–400 g; Charles River, Wilmington MA) were individually housed in an enriched environment (Nylabone® chews) on a 13/11-hour light-dark cycle (light 0600-2000). Animals were maintained on a restricted feeding schedule (15–20 g of standard chow available immediately after training/testing). All procedures were in accordance with NIH guidelines and were approved by the University of Wisconsin Institutional Animal Care and Use Committee.

### Behavioral Training/Testing

#### Training

Animals were trained in a T-maze delayed-non-match to position task as described previously (**Fig. 1E**; overall dim, 90 cm wide×65 cm long; runway dim 10 cm wide×10 cm high) [30]. Initial training was complete when animals entered the T-maze arm opposite from the last one visited for food rewards (chocolate chips 1.6 gm) delivered by the experimenter's hand with 90% accuracy on 10 trials (0 seconds delay, 1 session/day). Animals were then surgically implanted with recording electrodes and returned to *ad lib* feeding for the duration of recovery (7–10 days). Following recovery, training continued until animals performed two sessions of 41 trials at criterion of 90–100% correct for 2 consecutive days. Sessions were separated by 2 hours to minimize reward satiation/decreased motivation and carbohydrate-induced changes in cognitive function [86]. Importantly, no differences in performance existed between the first and second sessions. Although animals had learned the task, over time, performance in this task gradually improves at a given delay. To maintain baseline performance near 90% correct, the duration of the delay period was increased for each animal as necessary, ranging from 10–40 sec, and a new stable baseline was determined. Olfactory and visual cues were minimized by wiping the maze between each trial with 10% ethanol and lining the walls of the testing suite with black matt cloth. Masking white noise (60 db), measured at the intersection of the T (A-weighted; 2232, Brüel & Kjær Nærum Denmark), was generated from a speaker 2 meters above the center of the maze. During training sessions, animals were tethered to a dummy wire harness of identical weight and flexibly as the harness used for electrophysiological recording on testing days. After acclimation to the tether, animals showed no differences in maze performance or overt behaviors from prior reports [30].

#### Testing

On the morning of testing, an animal was placed in his home cage, on top of the T-maze, 2 hours before the first session began to allow the animal to habituate to the tether and the recording arena and allowed the experimenter to discriminate neural activity. Although animals had access to water and were able to freely move about their cage, during this period animals predominantly slept. The first session (Baseline), was conducted in an identical manner to prior testing days (41 trials, 60 db white noise; **Video S1**). During the second testing session of the day, presentation of the white noise (93 db) stressor was begun immediately prior to testing and presented continuously throughout the duration of the 41 trials. White noise as stressor has been shown previously to impair PFC-dependent functions in rats, monkeys, and humans [38], [48], [51], [57], [63] and activate the stress-related circuits within the brain as well as the hypothalamic-pituitary axis of rats [49]. Testing with noise stress was permitted at most 1/week.

### Surgery and Neural Data Collection

Under halothane anesthesia (Halocarbon Laboratories, River Edge, New Jersey; 1%–4% in air), animals were implanted bilaterally with linear electrode arrays (n = 8 electrodes/array; 250 µm separation; SB103, NB Labs, Dennison, TX) targeting layer V of the prelimbic region of the PFC (_pl_PFC) as previously described [52]. Electrode arrays contained 50 µm stainless-steel electrodes orientated in a rostral-caudal direction. Electrodes were attached to skull screws (MX-0080-16B-C, Small Parts, Inc.) with dental acrylic (Plastics One, Roanoke, Virginia), the wound was closed with wound clips (9 mm Autoclip; BD Diagnostic Systems, Sparks, Maryland), and animals were allowed to recover for 7–10 days.

On testing days, animals were brought into the T-maze testing room and tethered to the Multichannel electrophysiology Acquisition Processor (MAP, Plexon, Dallas, Texas). During the 2 hour habituation period, putative single “units” of the _pl_PFC were discriminated in real time using online template matching algorithms to preliminarily discriminate action potentials exhibiting a 3∶1 signal to noise ratio. Following discrimination of _pl_PFC units, animals remained tethered to recording hardware and the quality of the discrimination was monitored throughout the remainder of the day. During baseline and noise stress conditions, neural activity was simultaneously amplified, discriminated, time stamped, and recorded from these putative single units of the _pl_PFC as previously described [46], [52]. Additionally, video recordings were made of animal behavior during testing sessions (resolution = 0.0125 sec) with time-stamp overlays synchronized to the electrophysiological hardware. During the 2-hour inter-session interval, animals remained tethered and neuronal activity was monitored for drift in the quality of discrimination of action potentials.

### Histology

At the end of the study, animals were deeply anesthetized and cathodal current (60 µA) was passed across microwire pairs within a bundle for 45 sec. Animals were perfused with a 10% formalin + 5% potassium ferrocyanide solution that produced a Prussian blue reaction product at the electrode tip. Brains were removed and immersed in 10% formalin for 24 hr. Frozen 40-µm coronal sections were collected through the _pl_PFC and counterstained with Neutral Red. Representative placements of recording electrode bundles within the _pl_PFC are illustrated in **Fig. 1C**.

### Spike Train Analysis

#### Data pre-conditioning

After each day of recording, pre-established offline criteria were used to verify that waveforms assigned to each discriminated “unit” originated from a single neuron (**Fig. 1D**). These previously described criteria [46], [52] were based on unit waveform properties and spike train discharge patterns including: 1) variability of peak waveform voltage, 2) variability of waveform slope(s) from peak to peak, 3) separability of clustering of scattergram points from the waveform's first two principal components, and 4) refractory period evident in the spike train auto-correlegram. Neurons that met these criteria were further classified as “wide spike” (WS-type) or “narrow spike” (NS) to putatively identify large projection pyramidal neurons (WS-type) [53]. Essentially, the peak-peak (P-P) duration of waveforms from verified neurons were calculated. Neurons with P-P intervals greater than 200 µs were classified as WS-type neurons. Other cells classified as NS neurons (P-P intervals between 100–200 µs) or neurons not meeting either category were eliminated from further analyses. Lastly, _pl_PFC neuron action potential shape, neuron discharge pattern (inter-spike interval) and response properties were further examined to verify that neurons were not recorded across multiple recording sessions. Although our unpublished data indicate neurons recorded from multiple session days (separated by a week) occurs infrequently, if identified, analyses of data was limited to the first recording session of that neuron.

Behavioral events of the T-maze task were identified by visually scoring the time-stamped video recordings and manually entered into each neural recording data file. These events included 1) placement of the rat into the start box, 2) removal of the start gate, 3) rat reaching the branch point of the “T”, 4) the rat entering one of two goal arms (choice), 5) receipt of food reward, and 6) removal of the rat from the maze (**Fig. 1**). The intervals between these events were used to generate PETH's or as predictor variables included in the CI-GLM and defined respectively as the 1) Delay interval, 2) Run interval - running down the main arm of the maze, 3) Branch interval - orienting to the left/right arm, 4) Choice interval - complete entry into one arm, 5) Reward interval - consumption of reward, 6) Pickup interval - experimenter returning animal to start box. Additionally, each trial was further classified as a correct or incorrect trial, by the chosen spatial goal (i.e. left vs. right arm), and whether it occurred during the baseline or noise stress recording session. For analyses involving interaction terms included in the CI-GLM, we extended the delay to include the pickup interval in this analysis since the pickup also likely represents a segment of the delay period and that the maximal amount of data was needed to calculate the large number of interaction terms included in the CI-GLM. Additionally for analyses involving interaction terms, we defined the behavioral response interval as the group of contiguous behavioral intervals that include the Run, Branch, and Choice. Together, these behavioral response intervals represent the response to navigate the T-maze in an attempt to acquire a reward.

#### Data analyses

Spike train activity was characterized by either PETH analysis or by fitting conditional intensity functions to neural spike train data using generalized linear models [41]–[45] to describe the effects of stress on SHPD (NeuroExplorer, Nex Technologies, NC and custom Matlab functions; Mathworks, Natick, MA). These analyses were limited to correct trials, given that few error trials occurred during baseline testing sessions and reliable estimates of neuronal spiking activity were difficult to obtain for error trials.

T-maze task-related patterns of discharge for individual neurons were initially analyzed with trial-by-trial bin counts (5 ms bins) collected during baseline and noise stress conditions. For each recording session, trial bin counts corresponding to each behavioral interval within a trial were summarized as a temporally normalized PETH and quantified. Temporal normalization of PETH's was necessary because, across trials of a session each behavioral interval type (e.g. Run) was comprised of different durations of time. The mean discharge rate of a neuron, calculated for each behavioral interval during stress conditions, was then represented as percent of the baseline recording session discharge rate (first recording session of the day). For all recorded neurons, a one-way ANOVA analysis (TaskComponent – behavioral interval) was performed on these normalized PETH-generated data. False Discovery Rate (FDR)-corrected single sample T-tests were used to determine that discharge rates during each behavioral interval were significantly different from values of 100.

**Figure 6:**
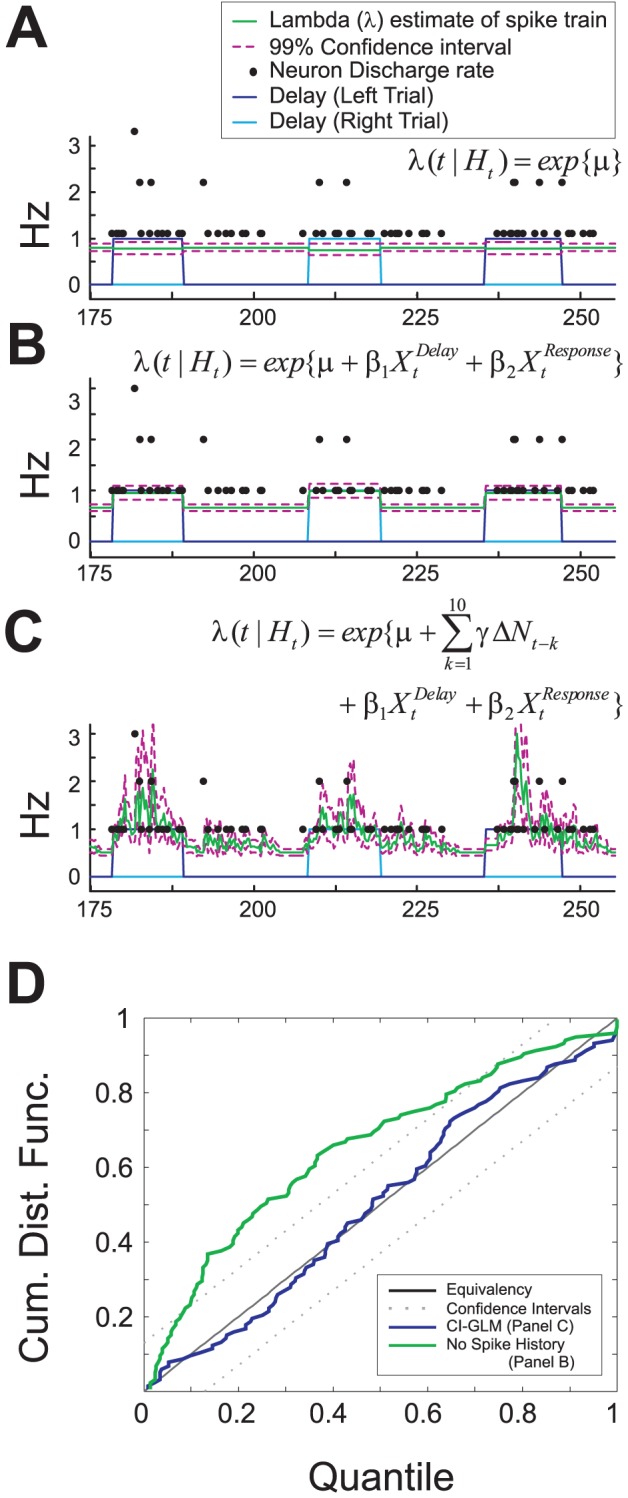
Characterization of generalized linear models of task-related activity. Plot of 80 seconds of spike train data, spanning three trials and fit with a GLM using (**A**) a homogeneous Poisson model, (**B**) an inhomogeneous Poisson model, and (**C**) an a conditional intensity model (Model 1b, during baseline conditions only). Spike counts of the original spike train are plotted with black dots against lambda (*λ*; green line with red confidence intervals). X-axis = experimental time. **D**) Kolmogorov–Smirnov (K-S) goodness-of-fit plot demonstrates that incorporation of spike history improves performance of the CI-GLM (blue vs. green line). The K-S plot of the final model (blue line; model from panel C) falls within equivalency confidence intervals of the K-S test (diagonal solid and dotted lines) for all quantiles, indicating that inclusion of spike history with behavioral intervals in the CI-GLM is critical to appropriately model _pl_PFC spiking activity. Inhomogeneous Poisson models using solely the behavioral states of the task overestimate neuron interspike intervals (green line; model from panel B). Models of neuronal activity (1–3; main text) also passed K-S goodness-of-fit tests (Fig. S3).

A CI-GLM was also used to directly analyze the effects of noise stress on T-maze task interval-related patterns of discharge for each neuron. The conditional intensity function 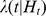 completely describes a neuronal point process [87]. Where 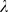 is the estimated generalization of the rate function of a Poisson process at time 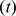 and 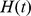 encompasses a number of covariates that the intensity function is conditioned upon. To fit the conditional intensity function to PFC neuron discharge, a Poisson - generalized linear model (GLM) framework was used [42]–[44], [55], [88]–[92]. During an evaluation step, we found that neither a homogeneous Poisson model (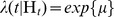; **Fig. 6A**) or an inhomogeneous Poisson model (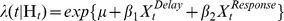; **Fig. 6B**) that included the intercept and T-maze behavior intervals as covariates could adequately fit the spike train data with a GLM. However, a conditional intensity model estimated as a function of spike history combined with each behavioral interval using the final model parameters (10^th^ order autoregressive filter and 250 ms binning) (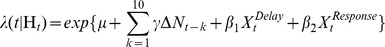 **Fig. 6C**) fit spike trains well, as determined by visual assessment and quantatively with a Kolmogorov–Smirnov (K-S) goodness-of-fit test (**Fig. 6D** **and Fig. S3**). The first model used to determine the overall effects of stress on SHPD was
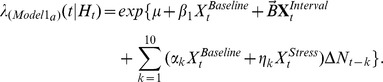
The conditional intensity function 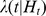 was predicted for each _pl_PFC neuron from a series of covariates where (*μ*) is the intercept of the equation representing the background level of activity. 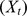 is a given manipulation (e.g. Baseline/Stress) for each sample in time (*t*). As such, the overall effect of the manipulation (Baseline/Stress) 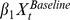 or each of the T-maze behavioral intervals 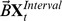 could be represented by these model covariates. *β* and *B* represent the fitted parameters for the manipulation covariate and matrix of behavioral interval covariates. Differences between left and right trials were not distinguished with this model. Spiking history during baseline or noise stress conditions 
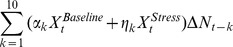
, is the change in the number of spiking events 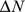 at one of 10 discretized time points in the past (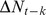). The fitted parameters for coefficients for this tenth order autoregressive process during baseline and stress are (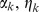). A more parsimonious expression of this model, 
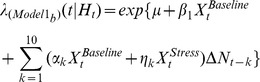
was also used to determine the overall effects of stress on SHPD. Given the animal always occupied one of the T-maze behavioral states 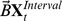 could be collapsed to simply 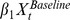. The deviance test (likelihood ratio test) was used to compare these nested models for each neuron.

An interaction between spike history and the behavioral components of the task was then determined with the following two models:
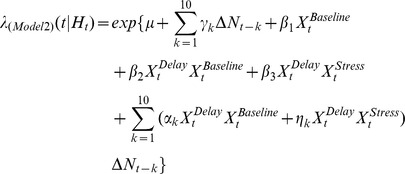
and
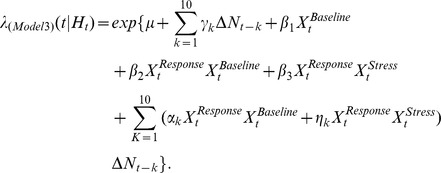
For these models, the main effects include the background level of activity (*μ*), the overall effect of spiking history 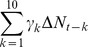, and the overall effect of the manipulation (Baseline/Stress; 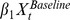). Additionally, the effect of baseline vs. noise stress for a given behavioral interval of the T-maze task (extended Delay, Response interval) was also modeled (e.g. 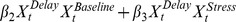) with 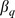 as the estimated weighting parameters. The interaction terms of Model 2 and 3 include the product of the autoregressive process, individual behavioral intervals of the T-maze task, and Baseline/Stress conditions represented by 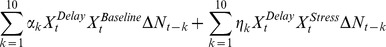, where 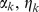 are the estimated weighting parameters of the interaction terms. Importantly, models 2 and 3 examine the interaction with the extended Delay period vs. Response period in separate models.

The degree to which the CI-GLM output (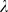) reflects the spiking activity of each _pl_PFC neuron was determined by visual assessment and quantatively with a Kolmogorov–Smirnov (K-S) goodness-of-fit test (**Fig. 6D**). Because the interaction models are not nested they could not be directly compared using the deviance test. However, a measure of “spiking gain”, equivalent to the rate-ratio (exponentiated covariate weights i.e. *β, α, η*) [54], representing the fold-change in predicted discharge activity given all other spiking activity occurring during the T-maze task was used to determine the effects of stress on SHPD. Statistical differences between the baseline recording session and noise stress were determined with a two way repeated measures ANOVA analysis (rmANOVA; Experimental Condition*Spike History Time) performed on SHPD gains. LSD post-hoc tests were used to make comparisons between individual groups. False Discovery Rate (FDR)-corrected single sample T-tests were used to determine that spiking rates or gains were significantly different from values of 1.0.

## Supporting Information

Figure S1 Stress-related changes in _pl_PFC neuron spike-history predicted discharge (SHPD) throughout baseline and acute stress conditions quantified using Model 1b. **A**) SHPD at different points back in time decay exponentially under baseline and stress conditions for the same ensemble of neurons used for Model1a. For this reduced model, which did not explicitly represent all behavioral intervals, stress also produced a significant reduction in SHPD gains (main effect) without significantly alter the decay of gains at any spike history time bin. **B**) Histogram plots of calculated GLM deviance for each neuron using Models 1a (top) and 1b (bottom). For the majority of neurons, the measure of deviance was significantly reduced by incorporating behavioral intervals into the model.
(EPS)

Figure S2 Action potential waveforms of 5 discriminated and validated _pl_PFC neurons and rejected electrical activity. Neuronal action potential waveforms and clusters in principal component space (inset) are replotted from Fig. 1E. Additionally, unsorted activity is included. Importantly, rejected activity includes both unsorted spiking activity and muscle artifacts including chewing. Waveform width = 450 µs.
(TIF)

Figure S3Kolmogorov–Smirnov test plots of _pl_PFC time-rescaled spike trains. Each panel represents K-S plots from each of six _pl_PFC neurons from three separate animals. The solid black diagonal line represents equivalency between the actual _pl_PFC spike train and 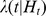 from the CI-GLM model. Dotted lines represent the 95% confidence intervals for equivalency measures. Inclusion of spiking history covariates into the model (blue line; Model 1a of main text) significantly improves 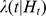 estimations from models that only include behavioral intervals (green line, model from Fig. 6B).(EPS)

Video S1 Video clip of electrophysiological recording while performing the T-maze task. Three trials are shown, a left-correct, a right-correct, and a right-incorrect. The green inset text denotes the animal ID, experimental time, and video frame number.
(MOV)
