# Supplementary figures and images for: Stress-Induced Impairment of a Working Memory Task: Role of Spiking Rate and Spiking History Predicted Discharge
Source: PLoS Comput Biol. 2012 Sep 13;8(9):e1002681. doi: 10.1371/journal.pcbi.1002681 (PMC3441423; doi:10.1371/journal.pcbi.1002681)

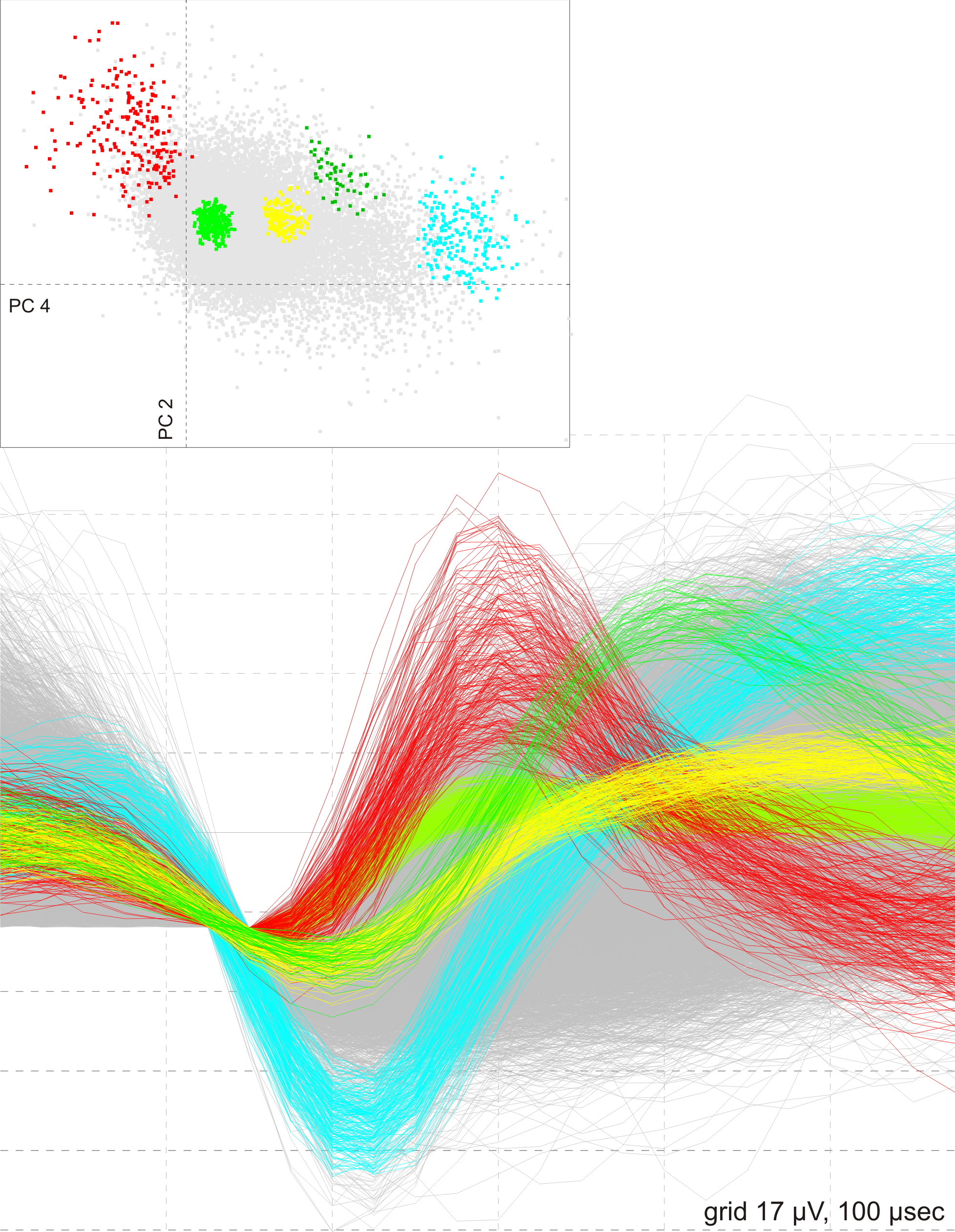

Supplement: Figure S2 — Action potential waveforms of 5 discriminated and validated plPFC neurons and rejected electrical activity. Neuronal action potential waveforms and clusters in principal component space (inset) are replotted from Fig. 1E. Additionally, unsorted activity is included. Importantly, rejected activity includes both unsorted spiking activity and muscle artifacts including chewing. Waveform width = 450 µs. (TIF) [file pcbi.1002681.s002.tif]
